# Exploration of the DARTable Genome- a Resource Enabling Data-Driven NAMs for Developmental and Reproductive Toxicity Prediction

**DOI:** 10.3389/ftox.2021.806311

**Published:** 2022-01-19

**Authors:** Elzbieta I. Janowska-Sejda, Yeyejide Adeleye, Richard A. Currie

**Affiliations:** Product Safety Early-Stage Research, Syngenta International Research Centre, Bracknell, United Kingdom

**Keywords:** AOP(s): adverse outcome pathway(s), DART: developmental and reproductive toxicity, NAMs: new approach methods, IATAs: integrated approaches to testing and assessment, bioinformatics, network analysis, HDG: human DARTable genome

## Abstract

The identification of developmental and reproductive toxicity (DART) is a critical component of toxicological evaluations of chemical safety. Adverse Outcome Pathways (AOPs) provide a framework to describe biological processes leading to a toxic effect and can provide insights in understanding the mechanisms underlying toxicological endpoints and aid the development of new approach methods (NAMs). Integrated approaches to testing and assessment (IATA) can be developed based on AOP knowledge and can serve as pragmatic approaches to chemical hazard characterization using NAMs. However, DART effects remain difficult to predict given the diversity of biological mechanisms operating during ontogenesis and consequently, the considerable number of potential molecular initiating events (MIEs) that might trigger a DART Adverse Outcome (DART AO). Consequently, two challenges that need to be overcome to create an AOP-based DART IATA are having sufficient knowledge of relevant biology and using this knowledge to determine the appropriate selection of cell systems that provide sufficient coverage of that biology. The wealth of modern biological and bioinformatics data can be used to provide this knowledge. Here we demonstrate the utility of bioinformatics analyses to address these questions. We integrated known DART MIEs with gene-developmental phenotype information to curate the hypothetical human DARTable genome (HDG, ∼5 k genes) which represents the comprehensive set of biomarkers for DART. Using network analysis of the human interactome, we show that HDG genes have distinct connectivity compared to other genes. HDG genes have higher node degree with lower neighborhood connectivity, betweenness centralities and average shortest path length. Therefore, HDG is highly connected to itself and to the wider network and not only to their local community. Also, by comparison with the Druggable Genome we show how the HDG can be prioritized to identify potential MIEs based on potential to interact with small molecules. We demonstrate how the HDG in combination with gene expression data can be used to select a panel of relevant cell lines (RD-1, OVCAR-3) for inclusion in an IATA and conclude that bioinformatic analyses can provide the necessary insights and serve as a resource for the development of a screening panel for a DART IATA.

## 1 Introduction

The identification of developmental and reproductive toxicity (DART) is a critical component of toxicological evaluations in the safety assessment of new chemicals, agrochemicals, and pharmaceuticals. Typically, this has required testing of chemicals *in vivo* using vertebrate species (usually rats and rabbits, but also sometimes mice, primates or latterly zebrafish) as models of humans. As a scientific field, toxicology is moving increasing towards using more modern scientific tools and understanding by applying so called new approach methods (NAMs). For instance, the concept of the adverse outcome pathway (AOP) (Ankley et al., 2010) organizes the required key events (KE) that underly adverse outcomes (AOs). Briefly, processes starting from the molecular initiating event (MIE) through additional KEs at the different levels of biological organization (biochemical, cellular, tissue, organ) to whole organism and population level responses are mapped to determine an AOP which could aid the development of NAMs by providing a focus on the relevant KEs that can be prioritized for method development. Integrated approaches to testing and assessment (IATAs) can be developed based on quantitative knowledge of the key event relationships (KERs) within the AOPs and so can serve as pragmatic science-based approaches to chemical hazard characterization. Next generation risk assessment frameworks have been proposed (Thomas et al., 2019) that use tiered testing strategies and the quantitative and mechanistic knowledge codified in AOPs as ways to identify points of departure to be used in risk assessments.

However, DART effects remain difficult to predict given the diversity of biological mechanisms operating during ontogenesis. Consequently, there are many potential MIEs that might trigger a DART AO. To build an effective IATA predictive of DART effects requires us to have a sufficiently complete understanding of the AOP Network (AOPN) that result in DART AOs. Identifying this DART AOPN is a key building block that provides the knowledge needed to create an IATA for use in next generation DART risk assessments. The identification of the molecular initiating events (MIEs) within this AOPN is therefore the first critical pieces of information to be discovered. A second is to show that the proposed tier 1 screens using omics and image based high-content methods provide sufficient biological coverage to detect these DART MIEs. If we knew this, then we could create a DART IATA that uses broad *in vitro* tier 1 screens to generate hypotheses for DART potential and then pragmatically focus higher tier testing to explore these hypotheses by identifying the target site exposure that is sufficient to quantitatively trigger the MIE.

Detailed information on our current understanding of biology and model systems is accessible through a wide variety of bioinformatics data sources. Consequently, it has been proposed that using appropriate bioinformatic analyses may aid the development of DART IATAs (Baker et al., 2018). Here we report how such an integrated bioinformatic analysis of gene-phenotype, MIE, human protein interactome and cell line mRNA expression data can provide solutions to aid DART IATA building. We intend these data to act as a resource for the community to aid in the design of experiments towards building a tier 1 screen in a DART IATA. The first solution aims to identify and suggest prioritization approaches for DART MIEs protein targets. The second solution is an approach to rationally select a panel of cell lines that could be used in an IATA to provide adequate coverage of the biology. We hypothesize an AOPN that results in developmental or reproductive defects in mammals. At a minimum, this hypothetical DART-AOPN must contain all gene products that an exogenous toxicant might bind with to trigger an MIE. In addition, it must also contain all genes and gene products that might have their abundance changed or activity altered in response to an exogenous toxicant. This comprehensive set of proteins that participate in MIEs or are the transcriptomic/proteomic KE biomarkers of DART effects we define for this paper as the hypothetical “human DARTable genome” (HDG) by analogy to the “Druggable Genome,” which is a comprehensive subset of genes that meet some specific criteria for potential to be drug targets (Finan et al., 2017). Baker et al. (2018) proposed a developmental toxicity ontology that codified the relationships between disparate data types that might be helpful in solving this problem. Indeed, it has been shown that some gene knockouts (KOs) phenocopy adverse events caused by chemicals (Deaton et al., 2019). Therefore, we hypothesize that by mining the bioinformatic information on developmental phenotypes caused by both chemicals and mouse gene knockout we could identify other hypothetical MIEs for DART effects. Furthermore, by analyzing the properties of these genes we can prioritize the HDG to identify the likely MIEs for DART. Once the HDG is identified, we demonstrate that it is possible to use baseline gene expression data to rationally select a panel of *in vitro* cell lines which provides adequate coverage of the biological pathways and HDG.

## 2 Materials and Methods

### 2.1 Curation and Evaluation of the Human DARTable Genome

The hypothetical HDG (∼5 k genes) was assembled by combining three different gene sets. Firstly, we identified a set of 123 genes (referred to as known MIE genes) associated with high confidence to DART endpoints. The MIEs were based mostly on the extraction of specific target proteins described by Wu et al. (2013). This list was then supplemented by a small number of additional targets based on an internal review of findings seen in pregnant preliminary dose setting studies that were conducted in rats or rabbits with Syngenta proprietary research compounds between 2005 and 2018 (personal communication). The criteria for inclusion in this supplemental list were that at least two chemicals with the same molecular target induced the same pattern of major malformations and either 1) the available evidence showed that the systemic blood concentration was sufficient to modulate the activity of that target at the lowest observed effect level for the developmental effects and/or 2) the chemicals were from different chemical series. When gene assignments were ambiguous, we expanded the gene list to include all members of a gene family. Because many teratogens are also embryo lethal at high dose levels, the second gene set was provided by the ∼1.6 k genes curated as pup/embryo lethal in the Deciphering Mechanisms of Developmental Disorders database (https:/dmdd.org.uk) (referred to as Embryo Lethal Genes). Finally, we extracted the ∼4 k genes that had been curated with the annotation “Developmental Disorders” by searching Diseases and Function in the Ingenuity Knowledge base (referred to as IPA Phenotype) using the Ingenuity Pathway Analysis tool (IPA) (QIAGEN Inc., https://www.qiagenbioinformatics.com/products/ingenuitypathway-analysis). All three gene sets used to compile the HDG in this paper were extracted using database versions existing in 2018. Rodent gene identifiers were mapped to the corresponding human orthologues using UniProt (UniProt Consortium, 2021) and the complete list of the HDG can be found in Supplementary File S1. The HDG was compared to the Human Druggable Genome (Finan et al., 2017) and overlaps between the genomes were visualized using Venny 2.1 (Oliveros, 2007).

The distribution of protein classes encoded by genes in the HDG was determined by performing over/underrepresentation analyses of Panther Superfamily Protein Classes (Mi, et al., 2020) using a Benjamini-Hochberg false discovery rate (FDR) threshold of *p* < 0.05. Differentially overrepresented (FDR corrected *p* < 0.05) Gene Ontology Biological Processes (GO-BPs) for the HGD were determined in ShinyGO (Xijin Ge et al., 2020) and were further clustered using MonaGO (Xin et al., 2020) to group similar GO-BP terms based on Resnik (Resnik, 1999) similarity (minimum of 2.7) between terms.

### 2.2 Network Analysis of the Human DARTable Genome

The HDG was mapped on to the Human Protein-Protein Interaction (HPPI) network from IntAct, downloaded on August 2021, (Orchard et al., 2013), and was visualized and analyzed using Cytoscape (Shannon et al., 2003). Duplicate edges and self-loops were removed from the network. The topological properties of each node within the human interactome (node degree, clustering coefficient, average shortest path length, betweenness centrality, closeness centrality, eccentricity, neighborhood connectivity and topological coefficient) were calculated and Kolmogorov–Smirnov nonparametric statistical test was performed to compare distributions of topological properties between HDG and non-HDG. Then, the subsequent analyses were performed on the largest connected component.

Communities (highly connected groups of nodes/genes usually involved in similar functions) within the first and largest connected component of the network were determined by Louvain clustering, a greedy agglomerative algorithm (Blondel, 2008), using NetworX Python. Each community was evaluated to determine over/under representation of MIE and DARTable nodes. Functional enrichment (GO-BP) for key communities was evaluated (FDR <0.05) GO-BP using ShinyGO (Xijin Ge et al., 2020) and each significant GO-BP term was clustered based on shared number of genes.

The functional cartography (community structure) analysis characterizes nodes according to their roles in each community (Guimerà and Nunes Amaral, 2005). Here, the analysis was performed for the communities detected via Louvain algorithm within the main connected component (CC) of the HPPI network. The cartography calculation depends on the following two properties: within-community module connectivity (z-normalized within community module degree) and participation coefficient (proportion of links a node has to members of other communities). Based on the region in a parameter space of z-score and participation coefficient, nodes were categorized as hubs and non-hubs and the five following categories were identified within the main component of the HPPI network: R1—ultra-peripheral node, R2—peripheral node, R3—non-hub connector node, R5—provincial hub and R6—connector hub (Supplementary File S4). The role of the nodes was determined using R software (version 4.1.1) Rnetcarto package. Following the identification of the nodes’ role within the first connected component of the HPPI network, the association of the node role (position) with DARTable effect was tested with the aid of chi-square test.

### 2.3 Using the DARTable Genome to Identify Relevant Cell Lines for Screening

A workflow was developed to query baseline RNAseq data of 934 human cancer cell lines from the Cancer Cell Line Encyclopedia (CCLE) (Barretina et al., 2012). After quality control, counts per gene were determined using FPKM (Fragments Per Kilobase of transcript per Million mapped reads) and then quantile normalized within biological replicates. Seven cell lines of interest were selected from CCLE for evaluation based on availability of historic L1000 data: human rhabdomyosarcoma (RD), human liver cancer cell line (HepG2), human ovarian carcinoma cell line (OVCAR-3), immortalized monocyte-like cell line (THP-1), human breast cancer cell line (MCF7), adenocarcinomic human alveolar basal epithelial cells (A549) and human renal adenocarcinoma (ACHN).

To determine the optimal cell line selection for use in chemical screening panel that would maximize coverage of the HDG whilst minimizing the cost, a procedure to select the most relevant cell line(s) was developed. Firstly, the 7 cell lines were ranked by the number of HDG genes they express that meet the selection criterion, which in this case, is illustrated using expression above the per gene average across all 7 cell lines. The top scoring cell line was then selected for the panel and the remaining cell lines were then re-ranked, based on how many HDG genes that met the selection criteria were not already covered by the cell line(s) already selected for the panel. Through such iterations, the optimal panel was selected to provide maximum gene coverage of the HDG using the minimum number of cell lines. Finally, to explore the biological coverage of the HDG provided by each cell line, HDG that were expressed in each selected cell line at above average levels were identified and evaluated for significantly (*p* < 0.05) enriched pathways compared to the Human Genome or the HDG using IPA (QIAGEN Inc., https://www.qiagenbioinformatics.com/products/ingenuitypathway-analysis).

## 3 Results

### 3.1 Curation of the Hypothetical Human DARTable Genome and Comparison to the Human Druggable Genome

**FIGURE 1:**
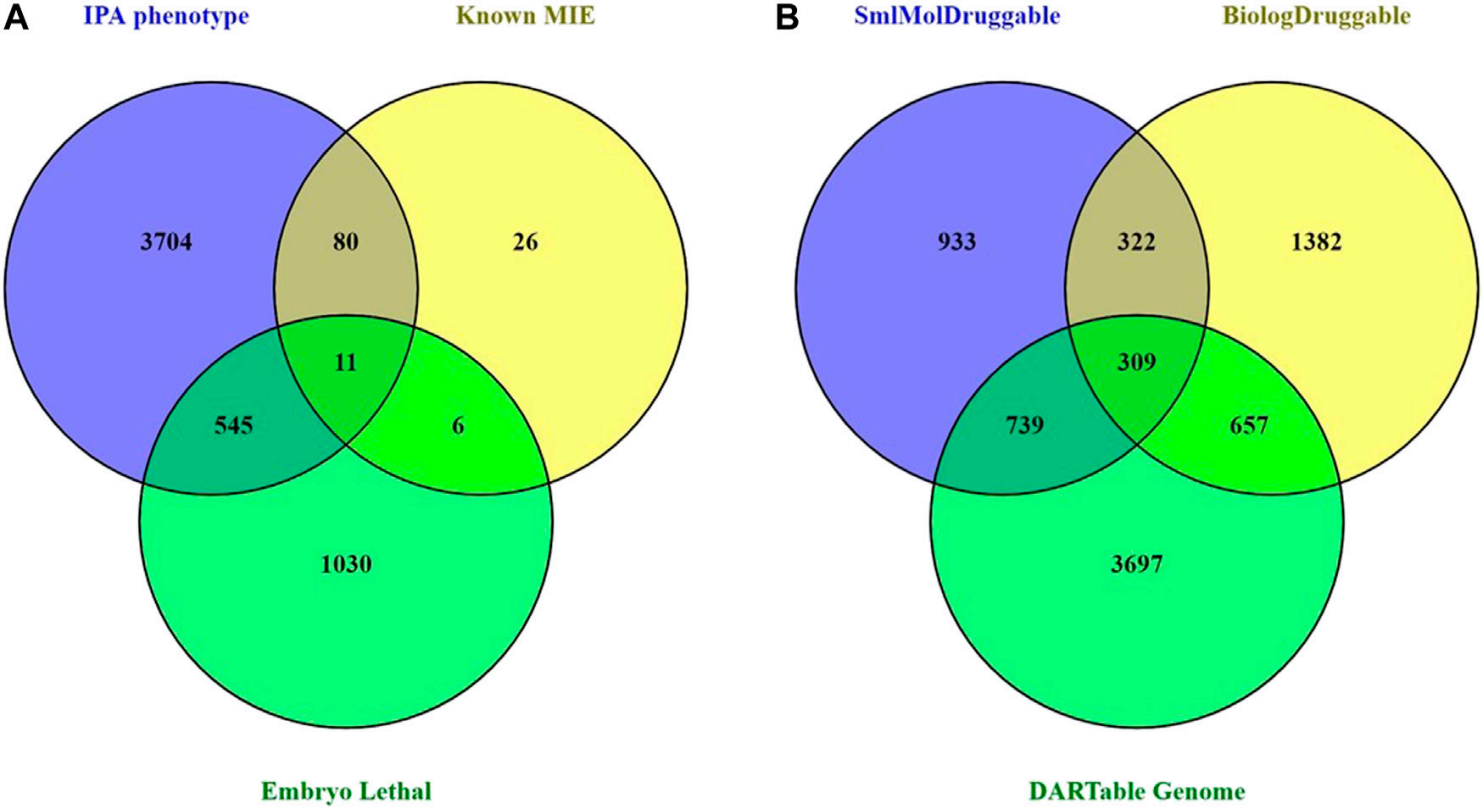
Curation and Analysis of DARTable Genome and Comparison to the Druggable Genome. **(A)** The overlap and differences between the memberships of each component source of the hypothetical DARTable genome was determined. Count represents a unique gene symbol. **(B)** The overlap between the hypothetical DARTable genome and the Druggable genomes from Finan et al., 2017 was determined.

To facilitate the creation of a data-driven DART IATA, we hypothesize an AOPN that includes a comprehensive set of MIEs that can result in any possible developmental or reproductive toxicity in a human. In addition, this hypothetical complete DART APON may also contain all genes and gene products that might have their abundance changed or activity altered in response to an exogenous toxicant. We term this comprehensive set of MIEs and transcriptional KE biomarkers as the hypothetical “human DARTable genome” (HDG). The first step in developing this complete DART AOPN is to identify the components of the HDG. We curated a high confidence subset of the HDG based on the known chemically induced DART MIEs from Wu et al., 2013. Further, we enhanced this set by adding a small number of additional MIE targets based on an internal review of unpublished findings seen in pregnant preliminary dose setting studies that were conducted in rats or rabbits with Syngenta proprietary research compounds between 2005 and 2018. We supplemented this MIE information with a bioinformatic selection of genes from IPA and DMDD.org that are known to have mutant or knockout alleles that cause either developmental phenotypes or to be embryonically lethal, respectively, in mice. In this way we created a comprehensive set of 5,402 genes known to be specifically important for development and reproduction (see Supplementary File S1 for the complete list). Figure 1A shows that the vast majority (97.7%) of this HDG is provided by the bioinformatic data based on phenotyping of mice mutants and knockouts.

As only 2.3% of the HDG was known to be a DART MIE, we next explored the relationship between the HDG and proteins with known, or potential, ability to bind with small molecule synthetic chemicals. Taking the Druggable Genome (Finan et al., 2017) as a proxy for these proteins, we determined the overlap between the HDG and the Druggable Genome (Figure 1B). There were 1705 (31.6%) members of the HDG that were also members of the Druggable Genome. Clearly a significant proportion of the HDG we identified may be binding partners for drug-like small molecule ligands and so should be considered as potential MIEs that could result in DART liability if their target site exposure is sufficiently high during development.

Interestingly, from the perspective of the Druggable Genome and the consequent DART safety risks for pharmaceuticals, this overlap represents 39.3% (1705/4,342) of the Druggable Genome. We next explored whether the small molecule interacting proteins were more likely to also be members of the HDG than biologic targets. The proportion of small molecule-tractable druggable genome targets that overlapped with the HDG rose to 45.5% (1,048/2,303) compared with only 36.2% (966/2,670) of the biological-tractable druggable genomes. This proportion rose to 49% (309/631) of the targets considered tractable to both small molecules and biologicals.

To further investigate members of the HDG and since 68% of the HDG did not overlap with the Druggable Genome, we explored which protein classes were represented in the HDG using Panther (Table 1). We found that gene specific transcriptional regulators, ligand gated ion channel and intracellular signaling molecules were very significantly overrepresented protein classes (*p* = 1.70E-10, 4.56E-05, 5.46E-05 respectively) whilst defense/immunity genes encoding defense/immunity proteins were significantly underrepresented (*p* = 1.05E-12). Furthermore, we identified that structural proteins in the extracellular matrix and cytoskeleton and histone modifying enzymes were also overrepresented (*p* = 3.00E-4, 9.72E-4, 8,38E-03 respectively).

**TABLE 1:** Proteins encoded by Genes in the Human DARTable Genome (HDG) by Protein Superfamily. Distribution of proteins encoded by genes in the HDG was determined by performing over (+) or underrepresentation (−) analyses of Panther Super Family Protein Classes (Mi, et al., 2020) using a false discovery rate (FDR) threshold of *p* < 0.05. Note that 1707 HDG genes were not classified by Panther.

Panther protein class name	FDR corrected p value	Over (+)/under (−) representation	Number of HDG genes (5,389)	Number of genes in human genome reference list (20,595)	Expected number of HGD genes	Fold enrichment
Defense/immunity protein	1.05E-12	−	47	498	130	0.36
Gene-specific transcriptional regulator	1.70E-10	+	483	1,280	335	1.44
Transporter/ligand-gated ion channel	4.56E-05	+	45	67	18	2.57
Intercellular signal molecule	5.46E-05	+	155	377	99	1.57
Protein modifying enzyme	2.98E-04	+	460	1,410	369	1.25
Extracellular matrix structural protein	3.00E-04	+	44	74	19	2.27
Non-receptor serine/threonine protein kinase	3.06E-04	+	133	328	86	1.55
Nucleic acid metabolism protein	3.21E-04	+	314	914	239	1.31
Cytoskeletal protein	9.72E-04	+	190	521	136	1.39
Histone modifying enzyme	8.38E-03	+	33	61	16	2.07
Serine/threonine protein kinase receptor	1.91E-02	+	11	12	3	3.5
Metabolite interconversion enzyme	2.93E-02	+	549	1843	482	1.14
Intermediate filament	3.37E-01	+	8	15	4	2.04
Calcium binding protein	5.93E-01	−	20	100	26	0.76
Transmembrane signal receptor	6.56E-01	−	271	1,100	288	0.94
Membrane traffic protein	7.15E-01	−	99	413	108	0.92
Cell adhesion molecule	7.23E-01	+	59	203	53	1.11
Chaperone	7.37E-01	+	54	187	49	1.1
Cell junction protein	8.41E-01	+	18	62	16	1.11
Translational protein	9.85E-01	−	86	335	88	0.98

### 3.2 Functional Analysis of the Human DARTable Genome

**FIGURE 2:**
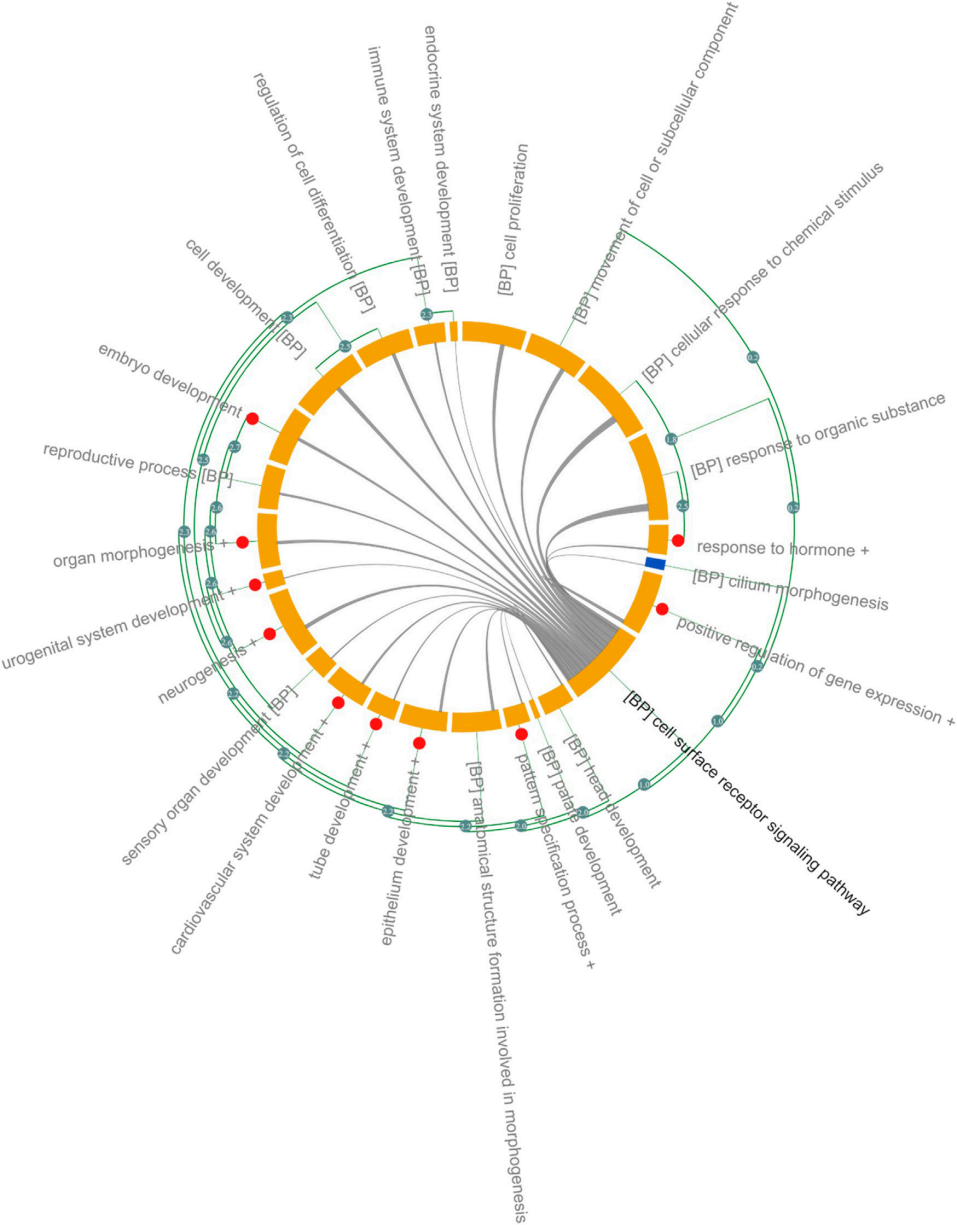
Gene Ontology Analysis of DARTable genome. Chord diagram (Xin et al., 2020) showing overrepresented (FDR corrected *p* < 0.05) GO-BP in the Human DARTable Genome from (A). GO-BP and were clustered based on Resnick similarity between terms. Each element (orange) on the diagram represents a GO-BP or clustered terms (Resnik similarity score>2.7) which are represented as red circles. The length of the element is proportional to the number of genes related to the GO-BP or cluster of GO-BPs. The green edges parallel to the main chord diagram on the outside show possible clusters between terms. The edges inside the diagram show shared genes between the GO-BPs and the width of the edges is proportional to the size of the overlap. In this example, GO-BP cell surface receptor signalling pathway (bolded) shares genes with sensory organ development, chemical response to stimulus and response to organic substance compared to endocrine system development. Percentage overlap between cell surface receptor signalling and other GO-BP can be found in Supplementary File S2.

Significantly enriched GO-BP is shown in Figure 2 and in Supplementary File S2. Due to the size and focus of HDG, ∼ 2 k GO-BP were significantly overrepresented therefore ranking GO-BP based on *p*-values, typically <0.05, would be meaningless. To reduce the complexity and aid interpretation, significant GO-BP were further clustered based on the Resnik similarity (>2.7) between GO terms. Cell surface receptor signaling pathway had the highest number of genes 1,288) and shared genes with almost all the GO-BP (Figure 2). Several genes within this group are associated with Wnt signaling and includes ligands (e.g., WNT1, WNT2, WNT3, WNT5) and receptors such as frizzled class receptors (FZD)1–10; receptors and ligands for Transforming Growth Factor Beta (TGFB) signaling: TGFBR and TGFB respectively and ligands (Fibroblast growth factors: FGF) and receptors (FGFR) associated with Ras signaling. Receptors (PTCH1, PTCH2 SMO), ligands (SHH and IHH) and transcription factors (GLI1, GLI2 and GLI3) of the Sonic Hedgehog (SHH) signaling pathway were also annotated with “cell surface receptor signaling pathway.” Transcription factors regulating the expression of genes associated with cell adhesion, proliferation, migration, differentiation as MYC, JUN, ELK1 and several cAMP responsive element binding proteins (CREB3L1) were significantly overrepresented in the “positive regulation of gene expression” cluster and 21% of genes overlap with cell surface receptor signaling pathway (Figure 2 and Supplementary File S2).

Eight GO-BPs (embryo development, embryonic morphogenesis, chordate embryonic development, *in utero* embryonic development, development of primary sexual characteristics, reproductive system development, embryonic organ development, embryonic placenta development) were clustered as “Embryo Development.” Genes within this category overlap with genes in the cell surface receptor signaling pathways (24%) and include members of the WNT and TGFB signaling pathway (Supplementary File S2). Several members of the homeobox family of transcription factors (HOXA, HOXB, HOXC and HOXD), retinoic acid (RA) receptors (RARA, RARB, RARG), the bone morphogenetic protein families (BMP2, BMP4 and BMP7) and matrix metalloproteinase (MMP) families (MMP14 and MMP) were associated with “Embryo Development” (Figure 2 and Supplementary File S2).

Organ morphogenesis, skeletal system, development, and morphogenesis were clustered into one group, named “Organ Morphogenesis,” which includes: development transcription factors such as the GATA family (GATA1, GATA2, GATA3, GATA4, GATA5, and GATA6), T-box transcription factors (TBX1, TBX2, TBX3, TBX20, and TBX5), head and neural crest derivatives expressed (HAND1 and HAND 2) and fork head box H1 (FOXH1). Members of the WNT, NOTCH and BMP family are also included in “Organ Morphogenesis” and 25% of genes within this category overlap with cell surface signaling pathways. The broad category, “Neurogenesis” (1 k genes), includes 3 GO-BPs: neurogenesis, central nervous system development and forebrain development. In addition to signaling pathways identified in cell surface receptor signaling pathways (overlap of 24%), receptors such as neuropilin (NRP1 and NRP2) and members of the semaphorin family (SEMEA3A, SEMA3F) were annotated with “Neurogenesis” (Figure 2 and Supplementary File S2). Other organ or anatomical specific GO-BPs such as immune system, sensory organ, urogenital, cardiovascular, head and platelet development were also significantly overrepresented.

Around 600 genes associated with “Reproductive Process” are overrepresented in the HDG and 15% overlap with cell surface signaling pathway. Genes encoding proteins involved in ovarian steroidogenesis such as luteinizing hormone beta polypeptide (LHB), luteinizing hormone/choriogonadotropin receptor (LHCGR), follicle stimulating hormone beta subunit (FSHB), follicle stimulating hormone receptor (FSHR) and CYPs cytochrome P450 family members such as CYP1B1, CYP11A1, CYP17A1, CYP19A1, and CYP2J2 were annotated with “Reproductive Process.”

### 3.3 Network Analysis of the Human DARTable Genome

A HPPI network used in this analysis consists of 20,922 nodes and 29,4922 edges. Nearly 94%, 5,074 genes, of HDG were mapped into HPPI including 112 (91%) MIE genes. 57 CCs were identified within the network, whereas the main component comprises of 99% of the total number of the nodes in the network (20,793 nodes) and 5,069 HDG genes are also present in this component. The nodes that are not connected (so-called orphan nodes) were also identified within the network and 4 out of 19 orphan nodes are DARTable genes: MIOX, CRYGD, APOBEC2, and LHCGR.

### 3.4 Comparison of Topological Properties of DARTable and Non-DARTable Nodes in the Network

**FIGURE 3:**
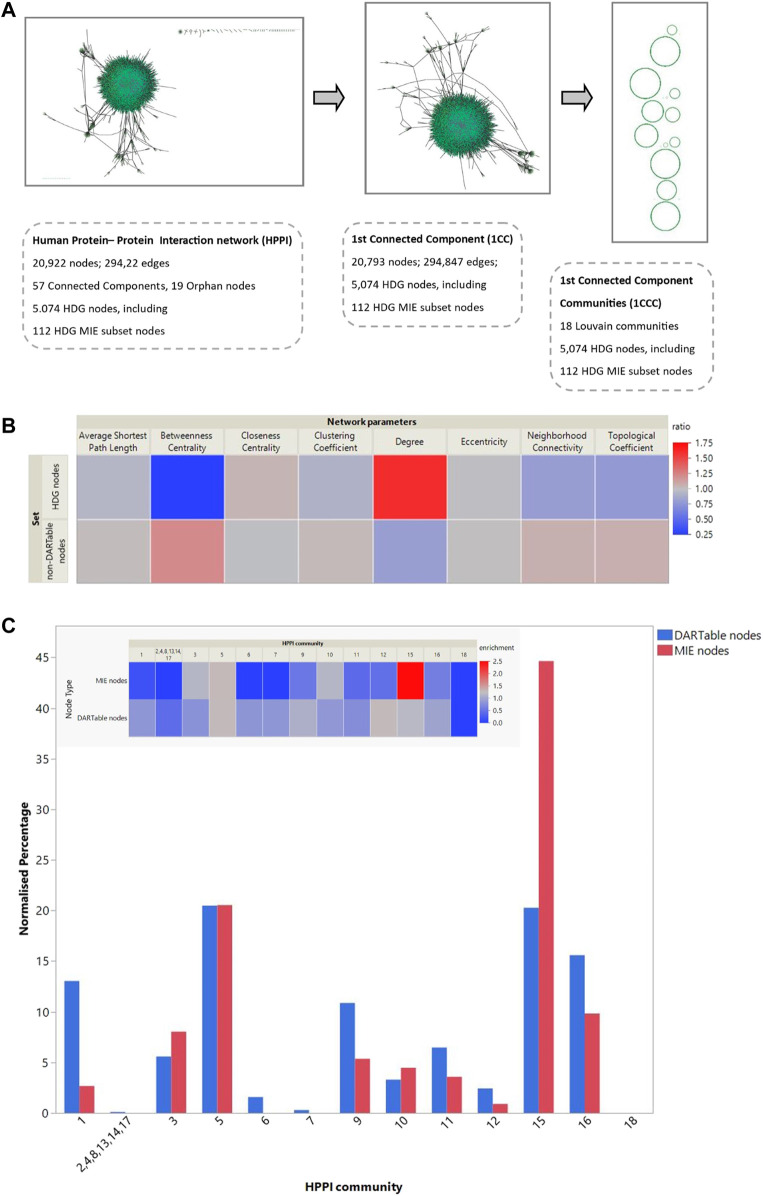
Network analysis of the DARTable genome. **(A)**. Network analysis: Using the human protein interactome information we created a network with 20,922 nodes and 294,922 edges, consisting of 57 connected components. The HDG mapped to 5,074 nodes in this network. The high confidence MIE subset mapped to 112 nodes. We extracted the largest connected component of the HPPI for further analysis. This retained 20,793 nodes and 294,847 edges from the full HPPI and contained 5,069 HDG nodes, including all 112 that map to the full HPPI. We performed Louvain clustering to identify 18 communities within this largest connected community and mapped the HDG and the known MIE subset to these communities. **(B)**. We calculated the average topological properties for the entire HPPI network, the non-HDG and the HDG subset of nodes (Sets 1,2 and 3, respectively–see Table 2 for more details). **(B)** shows the topological properties of both the HDG and non-HDG relative to the complete network where topological properties in red indicate an increase compared to the whole network and those in blue a decrease. **(C)**. Part C shows the percentage of the entire HPPI, the HDG and its MIE subset that maps to each of these communities. The inset heatmap shows the over- and under-representation of the HDG subsets relative to the HPPI.

We investigated if there were differences in network properties between HDG compared to other nodes (non-DARTable nodes). In this analysis we concentrate on all CCs of the HPPI network and topological properties of each node were calculated. Then, we defined 3 sets of nodes such as Set1: all nodes in HPPI, Set2: only nodes that are non-DARTable genes (nodes that are not part of HDG) and Set3: only nodes that are DARTable genes (analogically, nodes that are part of HDG). For each set of nodes, the average value of network parameters was calculated (Table 2). While comparing the average values for each calculated network parameter between Set2 and Set3 (Figure 3B; Table 2), we observed much higher node degree values for DARTable nodes. This also explains the slightly smaller value for the average shortest path length parameter for DARTable nodes. On the other hand, slightly higher value of clustering coefficient within non-DARTable nodes might suggest that DARTable nodes are less likely to create tightly knit groups, which is also visible while comparing neighborhood connectivity values between the two sets. Furthermore, distribution of each parameter was compared between Set2 and Set3 by conducting non-parametric statistical test, namely the Kolmogorov-Smirnov test (KS test). The KS test confirmed significant difference (*p* < 2.2e -16) between Set2 and Set3 for each tested network parameters.

**TABLE 2:** Topological properties of nodes in the network. Table 2 shows the average calculated topological properties of each node in the network. Three sets of nodes were determined: Set1: all nodes in HPPI, Set2: only nodes that are non-DARTable genes (nodes that are not part of HDG) and Set3: only nodes that are DARTable genes (analogically, nodes that are part of HDG). Value in brackets indicates number of nodes in each set.

Network parameters	Set1: HPPI all nodes (20,922)	Set2: HPPI Non-DARTable nodes (15,848)	Set3: HPPI DARTable nodes (5,074)
Average Shortest Path Length	3.3308	3.3901	3.1454
Betweenness Centrality	0.0009	0.0011	0.0002
Closeness Centrality	0.3069	0.3025	0.3207
Clustering Coefficient	0.0939	0.0965	0.0858
Degree	28.1925	22.6400	45.5351
Eccentricity	10.5788	10.6139	10.4693
Neighborhood Connectivity	186.9262	198.9715	149.3040
Topological Coefficient	0.1239	0.1328	0.0960

### 3.5 Community Structure Detection and Analysis of the Network

As the majority of the HDG is represented by the largest CC of HPPI network, this analysis concentrates on the main CC of Human Interactome (Figure 3A). In total 18 communities (modules) were identified. The resultant communities were found to have an uneven community-size distribution (Figure 3C). DARTable nodes are present in a larger part of communities, whereas MIE nodes are represented by half of the communities detected. Of interest here are communities 1, 5 and 15, where we observe a substantial overrepresentation of DARTable and MIE nodes (communities 5 and 15) and underrepresentation of MIE nodes (Community 1). This is especially visible in community 15, where MIE nodes are significantly enriched (Figure 2C). To further explore the roles of these communities we performed GO over-representation analysis (Table 3, Supplementary File S3). Significant GO-BP terms in Community 1 are broadly associated with transcription, keratinization, and embryo development, whereas Community 5 is enriched with intracellular signaling, cell migration and the cell cycle, and Community 15 is largely represented by transmembrane and ion transport, chemical homeostasis, GPCR signaling and purine nucleotide metabolic process.

**TABLE 3:** Functional annotation analysis of community clusters identified from network analysis. Communities 1, 5 and 15, were identified as key clusters within the Human Protein-Protein Interactome that was overrepresented with DARTable and MIE genes (Figure 3). Genes from each community was evaluated for GO-BP overrepresentation (FDR<0.05). Key over-represented GB-BP are reported in the table and the gene lists can be found in Supplementary File S3. The Count is the number of genes in the Community annotated with the GO-BP, % is the percentage of input genes annotated with the term compared to the annotated genes with the term. Fold enrichment is the ratio between the background frequency of total genes annotated to that term to the input gene list for the term.

Community	Term	FDR	Count	%	Fold enrichment
Community 1	Regulation of transcription, DNA-templated	3.31E-48	742	27.6	1.61
	Keratinization	1.13E-05	23	0.9	3.61
	Cell fate commitment	4.27E-03	55	2.0	1.77
	Embryo development	1.26E-02	162	6.0	1.33
Community 5	Intracellular signal transduction	5.35E-75	740	26.2	1.85
	Phosphorylation	2.27E-69	642	22.7	1.91
	Cell cycle	1.41E-42	463	16.4	1.84
	Cell projection organization	5.20E-39	378	13.4	1.93
	Cell migration	3.97E-26	319	11.3	1.79
	Cilium organization	7.05E-17	89	3.2	2.60
Community 15	Transmembrane transport	1.80E-74	503	15.0	2.19
	Ion transport	5.09E-72	532	15.9	2.10
	Chemical homeostasis	6.68E-35	348	10.4	1.91
	Lipid metabolic process	1.48E-27	392	11.7	1.70
	Inorganic ion transmembrane transport	2.55E-27	250	7.5	1.99
	Sphingolipid biosynthetic process	5.63E-16	56	1.7	3.29
	Protein exit from endoplasmic reticulum	6.42E-10	28	0.8	3.93
	GPCR signaling pathway, coupled to cyclic nucleotide second messenger	2.31E-07	68	2.0	2.03
	Cyclic purine nucleotide metabolic process	1.46E-06	54	1.6	2.13

Whilst comparing the node associated with DARTable phenotype to the node role in the network, we identified DARTable nodes to be highly represented by non-hub connector nodes (R3) with half of its links within the community and connector hub nodes (R6) with many links to other communities and at least of half of its links within-community (Supplementary File S4). Furthermore, chi-square test of association confirmed previous finding that DARTable nodes are more likely to form tight groups with many links to other communities. The null hypothesis stating that there is no association between the node position in the network and its DARTable properties was rejected (χ2 = 1,236.99, critical value = 7.82, *p* < 0.00001).

### 3.6 Using the DARTable Genome to Rationally Identify the Relevant Cell Lines for a DART Safety Panel

**FIGURE 4:**
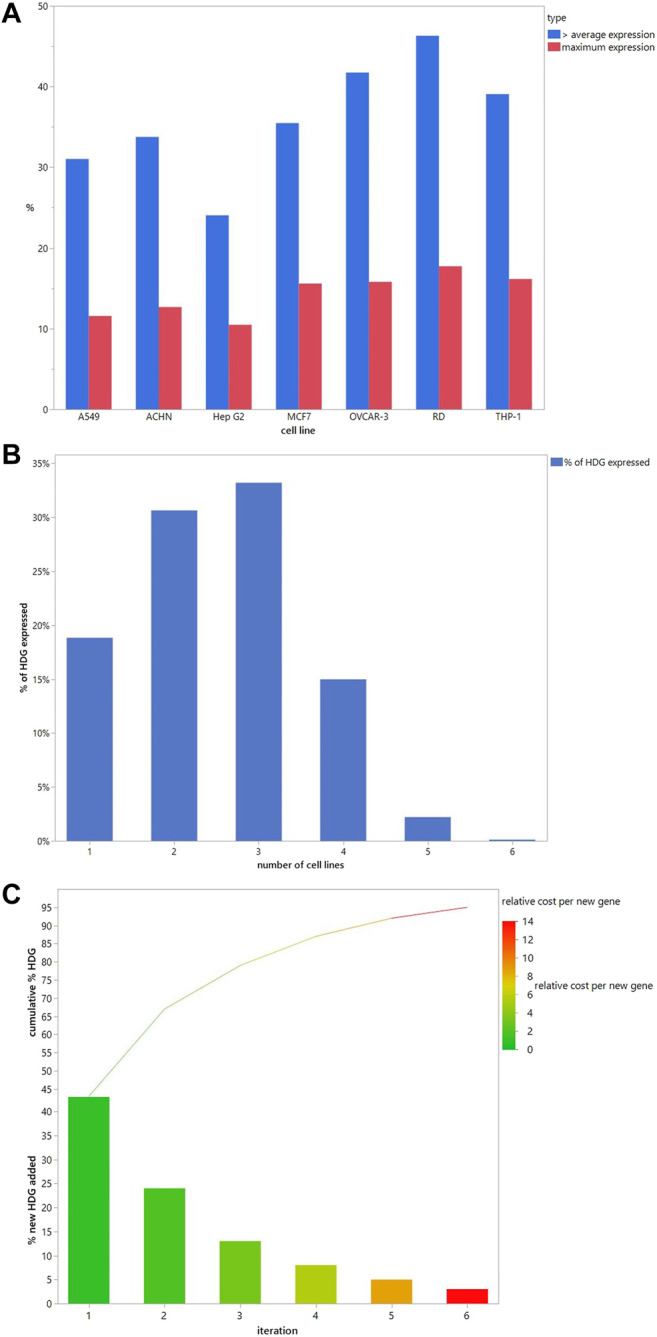
Cell line selection to cover the HDG. **(A)**. The number of HDG members each cell line contributes individually when the threshold is above the average per gene expression (blue) or has maximal expression (red). **(B)**. Percentage of HDG members that are expressed above the average per gene expression in the indicated number of different cell lines in the panel. **(C)**. When a cell line is iteratively added to the panel based on adding the largest number of new HDG members with above average per gene expression then the % new HDG members added (bars) and the running cumulative % HDG (line) increase as shown. Cell lines selected and the numbers added are in Table 4. Bars are colour coded to illustrate the increasing relative cost of each new HDG member added compared to the cost in iteration 1, which was set at 1.

The ideal DART safety screening panel would provide sufficiently complete coverage of all the known and potential MIEs in the HDG. Sufficiency of coverage would have to be defined based on the purpose. But it is conceivable that project de-risking during the research and development phases of new chemical invention may balance lower coverage to improve the cost-effectiveness for screening many compounds. Whereas the use as part of a weight of evidence to support regulatory DART risk assessments may require complete coverage of the HDG. To explore the rational selection of cell lines for coverage of the HDG, we investigated the basal expression of seven human cancer cell lines that have been used to generate transcriptomics signatures. We explored how many HDG genes were expressed at the maximum level and at greater than the per gene average expression level across the cell lines. Of the selected cell lines, a high proportion of the HDG was expressed at greater than the average expression level in at least 1 cell line (5,032 or 93.2%). Figure 4A shows the percentage of the HDG that each cell line expresses. There is considerable variation between cell lines from 31% with above average expression in A549 cells to 46% in RD cells at greater than the per gene average expression level. To explore the redundancy between the cell lines, Figure 4B shows the percentage of the HDG that are expressed across multiple cell lines. Interestingly in this panel of seven lines, 19% of the HDG is expressed at greater than per gene average in only a single cell type. Nevertheless 81% of the HDG was expressed in two or more cell lines (Figure 4B) and so the rational selection of a DART safety screening panel will require the selection of the optimal subset of the cell lines to meet the risk assessment need.

To build an optimal panel we iteratively selected the cell line that adds the largest number of new HDG genes above the per gene average expression. Selecting just 2 cell lines (RD and then OVCAR-3) covers 67% of the HDG (Figure 4C). Interestingly choosing this pair significantly increases coverage compared to two commonly used cell types (MCF7 and HepG2 combined only covers 51% of HDG) demonstrating that this selection method can improve relevant biological coverage. Continuing to select more cell lines in subsequent iterations adds a decreasing percentage of additional HDG genes per cell line. Consequently, more complete coverage comes at an increasing cost per additional HDG gene added. Assuming the cost scales with each cell line added, by iteration six, each additional gene will cost 14.5 times more than the first set (Figure 4C). Nevertheless, this extra cost should be weighed against the potential for increased sensitivity for the bulk of the HDG due to the redundancy of expression between cell lines (Figure 4B).

**FIGURE 5:**
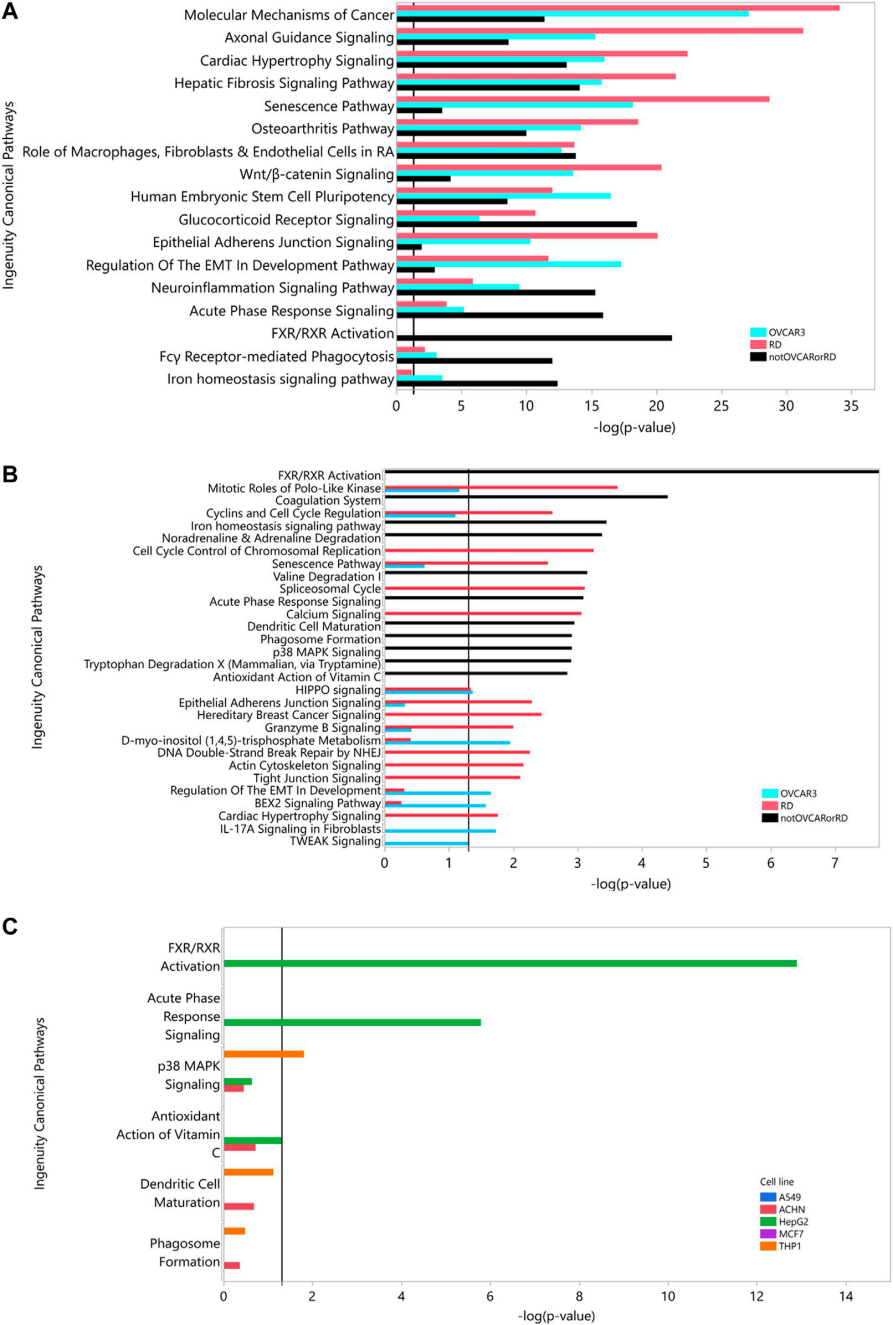
Pathway Analysis: Using the DARTable Genome to identify the relevant cell lines. **(A,B)**: Pathways significantly overrepresented for DARTable genes expressed above average in the top 2 cell lines (RD and OVCAR-3). DARTable genes expressed in RD (red) and OVCAR-3 (blue) and DARTable genes that were not in expressed in either cell lines were used as inputs (black). Pathways significantly enriched [*p* < 0.05 or –log(*p*) > 1.3], indicated by vertical black line, were identified after comparison to either the human genome **(A)** or the DARTable genome **(B)**. **(C)** Enrichment analysis of the 5 pathways that were not represented in the top 2 cell lines (RD and OVCAR-3). Enrichment analysis was performed using data from the other cell lines: A549, ACHN, HepG2, MCF7 and THP-1 pathways significantly enriched [*p* < 0.05 or –log(*p*) > 1.3], indicated by vertical black line, were identified after comparison to the DARTable genome.

Table 4 shows that at iteration three the top 2 cell lines add similar number of genes (THP-1, MCF7 add an additional 650, 630 genes respectively) and the remaining three lines add approximately the same number (∼530). Criteria other than just adding the largest number of HDG gene members may be more appropriate than just adding the numerically largest contribution. To explore the utility of selecting based on increased biological coverage we evaluated three gene lists for pathway enrichment analysis: DARTable genes that were expressed above average in RD and/or OVCAR-3 and DARTable genes that were not significantly enriched in either cell lines. Pathways associated with developmental defects such as altered differentiation, cell proliferation and motility, and developmental signaling such as WNT were significantly enriched in both RD and OVCAR-3, when compared to the human genome, suggesting the DARTable genome captures the key process for DART (Figure 5). Notably, the FXR/RXR (retinoid X receptor) pathway was not enriched in either OVCAR-3 or RD (Figure 5A). As a proportion of the HDG, signaling pathways such as HIPPO were enriched in OVCAR3 and RD; the FXR/RXR pathway and other immune response pathways were not significantly enriched (Figure 4B). To determine if the 6 pathways underrepresented (as a proportion of the HDG) in RD or OVCAR3 were enriched in the other 5 cell lines, DARTable genes that were expressed above average in MCF7, HEPG2, THP-1, ACHN and A549 were selected for pathway enrichment analysis (Figure 5C). The FXR/RXR activation, acute phase signaling response and antioxidant action of vitamin C were significantly enriched [−log(*p*) 12.9, 5.70 and 1.31 respectively] in the HepG2 cell line (Figure 5C). The p38 MAPK signaling pathway was significantly enriched in the THP-1 cell line only (−logp 1.8). Dendritic cell and phagosome maturation pathways were not significantly overrepresented in any cell line; however, these pathways were significantly overrepresented when genes from 5 cell lines were combined (Figure 5B).

**TABLE 4:** Using the DARTable Genome to Identify the Relevant Cell lines. Seven cell lines were ranked by the number of HDG genes they express that meet the selection criterion. The top scoring cell line was then selected for the panel and the remaining cell lines were then re-ranked, based on how many HDG genes that met the selection criteria were not already covered by the cell line(s) already selected for the panel. For each cell line in each iteration, we show the number of additional HDG with above per average gene expression that cell line would add and the cell lines ranking in parentheses.

Cell line	Iteration					
	1	2	3	4	5	6
RD	2,330 (1)					
OVCAR-3	2,100 (2)	1,197 (1)				
THP-1	1966 (3)	1,022 (3)	650 (1)			
A549	1,562 (6)	918 (4)	531 (=3)	388 (1)		
Hep G2	1,210 (7)	736 (6)	531 (=3)	369 (3)	248 (1)	
MCF7	1785 (4)	1,128 (2)	630 (2)	386 (2)	229 (2)	148 (1)
ACHN	1,699 (5)	902 (5)	529 (5)	361 (4)	159 (3)	106 (2)

## 4 Discussion

It is desirable to be able to rationally design an IATA for the prediction of DART. However, the complexity of the biological processes used to build an organism represents a daunting prospect for NAM developers and hopes for regulatory use. Although some mechanisms of DART have been identified (Wu et al., 2013), it is not certain that they provide adequate coverage of all the possible relevant biological mechanisms. The use of bioinformatics to maximize available biochemical data to enable the development and evaluation of NAMs, usually *via* the curation of AOPs, has been proposed (Carusi et al., 2018). One of the main challenges in developing NAMs for complex endpoints such as DART is understanding and mapping the molecular mechanisms underlining the toxicological endpoint of interest. Here we have determined the HDG using established DART MIEs, KO Lethal Genes and KO Developmental Phenotype Genes. This curated list of ∼5 k potential molecular targets in the HDG is a key resource to aid the research and development of NAMS for DART by maximizing the use of publicly available data and permitting maximal coverage based on our current understanding and information on developmental biology.

We defined the HDG as the set of genes the products of which may be MIEs or KE biomarkers in the AOPN that results in DART. Functional enrichment and network analysis of the HDG enables us to map out and understand the processes and pathways that interact to facilitate normal embryonic development, cellular organization, cell proliferation, organ, and tissue function and to determine when these processes are disrupted in DART. We identified known signaling processes/pathways and transcription factors that are key (sometimes in cross talk) to normal embryonic development and adult tissue homeostasis which as WNT, TGFB, FGF, RA, and SHH (Lavery et al., 2008; Liu et al., 2012; Tanaka et al., 2014; Steinhart and Angers, 2018; El Shahawy et al., 2019) and genetic disruption of these pathways can lead to developmental defects such as, autosomal recessive disorder Tetra-Amelia (loss of limbs) (Luo et al., 2007). In addition to genetic development defects, members of SHH, TGFB and RA signaling pathways have been proposed as potential biomarkers for embryotoxicity (Sipes et al., 2011; Piersma, et al., 2017). Broadly, activation of these pathways via ligand binding to receptors leads to the activation of transcription factors that regulate the expression of key genes associated with cell proliferation, tissue patterning, cell adhesion, organ size, differentiation. For example, activation of the WNT pathway via ligand binding to receptors such as FZD leads to activation of transcription factor such as beta catenin (*via* messenger proteins) to regulate the expression of key genes associated with proliferation, differentiation, transformation, and adhesion (Steinhart and Angers, 2018). On an organism/organ level, the WNT pathway is associated with embryonic development (Steinhart and Angers, 2018), neural cell differentiation from stem cells (Kondo et al., 2011; Piersma et al., 2017) and early-stage promotion of cardiogenesis (Bruneau, 2013).

In addition to cell signaling pathways, genes encoding transcription factors were also significantly overrepresented in the HDG and was corroborated by the network community analysis. Several transcription factors linked to activation of WNT, NOTCH, TGFB pathways were significantly overrepresented in the HDG. For example, the HOX family of transcription factors are expressed and regulated in embryonic development in mice and deletion of the HoxA cluster is embryonically lethal in mice (Kmita et al., 2005). Other transcription factor families such as GATA which is involved in regulating erythroid development in fetus (Crispino and Horwitz, 2017) and TBX (1–5) which are expressed in multiple tissues during embryogenesis have been associated with ear defects and craniofacial abnormalities in humans (Arnold et al., 2006) were present in the HDG.

Interestingly, network analysis revealed notable differences between the DARTable and non-DARTable nodes within the network. DARTable genes are more highly connected with a lower neighborhood connectivity than the other genes in the network and they exist in regions within the network. The betweenness centralities and average shortest path length are lower, making them highly connected to each other and to the wider network but not only to their local community. Conversely, proteins that are not part of HDG are characterized by lower degree and their role is associated to their own modules in the network. Similar findings were observed in the previous study (Piñero et al., 2018) for Proteins Associated to Drug Toxicity. This suggests that DARTable genes appear to be coordinators and regulators of biological processes and could connect different modules within the network which would be key characteristics of transcription factors and cell signaling.

We propose that the HDG could be a key resource in better understanding the underlying biology of DART and could shed new light on new avenues for investigative toxicology, NAM development, and target prioritization. Here we demonstrate two potential uses of the HDG: screening tool development and MIE characterization and development.

During the development of an *in vitro* screening tool, the selection of the most appropriate cell line(s) to recapitulate the *in vivo* biology is a significant challenge of using non-animal approaches for risk assessment. Complex endpoints like DART further complicate the challenge of integrating responses across multiple tissues and organs. Whilst organotypic models may address these issues, there are difficulties surrounding ease of use, cost, and lack of throughput. Increasingly the use of rapid high content *in vitro* assays, for example transcriptomics using technologies such as L1000 and TempoSeq or cell painting (Lamb et al., 2006; Bray et al., 2016), are being used to aid the identification of MIEs. However, the available data suggests that a prerequisite for being able to detect an MIE signature in such experiments is the baseline expression of the target gene in the cell line (Baillif et al., 2020). Data-driven approaches for unbiased cell line selection have been proposed (Thul et al., 2017) where cell lines can be clustered based on their transcriptional profiles and representative cell lines from each cluster are selected to ensure biological coverage and diversity of response. However, this approach does not necessarily ensure that the key processes of interested are represented. Here, by using a combination of prior knowledge of the potential molecular drivers of the adverse outcome (the HDG) and basal transcriptional profiles, we determined the minimum number of cell lines to select for subsequent studies that are optimized for biological coverage of DART. We initially identified that 2 cell lines, RD, and OVCAR-3, covered 67% of the DARTable genome and subsequent addition of more cell lines would improve the coverage of DARTable genes. However, this comes with a penalty of diminishing returns, as the cost of adding each subsequent cell line increases linearly whilst adding an ever-smaller proportion of the HDG. Using the HDG, we prioritized HepG2 as the additional cell line because the RXR signaling pathway, disruption of which can cause developmental defects, was not enriched in OVCAR-3 or RD but in was in HepG2 which is included in the panel. This approach could be used for any list of genes/pathways/processes that are key to the toxicological endpoints to aid cell line selection.

Our cell line selection approach focused on baseline expression to build a panel with appropriate biological coverage. However, the increasing availability of large datasets of gene expression data in multiple cell lines after genetic manipulation or chemical treatment (Subramanian et al., 2017) raises an intriguing possible avenue for future extensions of this work that address the question: is it possible to reduce the cell line coverage requirements (or conversely, increase the biological coverage per cell line) by identifying treatments that induce HDG members that are not basally expressed in that cell line? It is possible that modifications of the approach we describe here, when applied to the large number of possible cell line/treatment combinations, will permit us to identify greater biological coverage from a smaller number of cell lines.

Although we can be confident that the ∼5 k genes in the HDG are associated with DART phenotypes, it is not clear how many are potential MIEs. How can we determine what makes a member of the HDG a good candidate to be an MIE, as opposed to a biomarker of the activation of an MIE? We approached this in two ways: by exploring the potential to be a “receptor” for a xenobiotic, and by exploring the correct biological properties to trigger downstream consequences.

To participate in an MIE, by definition, a gene product must be able to bind to a xenobiotic. Consequently, we used the Druggable Genome as a surrogate for proteins that are likely have binding pockets that permit binding of small molecule xenobiotics and then alter their function. We showed that 31% of the HDG was also in the wider Druggable Genome as described by Finan et al., 2017. This information suggests that a significant fraction of the hypothetical HDG may not act as an MIE to a chemical stimulus because they are members of protein classes that are not known to be “ligand-able” by the known chemotypes that have been evaluated to date. Consequently, building a DART screening panel that focuses on the 1705 HDG genes targets that overlap with the Druggable Genome might be an appropriate pragmatic starting point. Furthermore, these targets are more likely to have known model chemicals that bind to them, published data in high content drug screens, and have published toxicology and pharmacokinetic data that in combination will be required to determine whether a potential MIE is in fact quantitatively able to be an MIE.

An interesting corollary to our analysis is that a considerable minority of the Druggable Genome may have a theoretical DART safety liability. This proportion is higher for the small molecule Druggable Genome than it is for the biological druggable genome as defined by Finan et al., 2017. Obviously, in clinical practice for this to be realized depends on the safety margin being inadequate to prevent DART effects. Nevertheless, this does suggest that a shift to novel targets accessible via biological drugs are less likely to have DART liability than the small molecules targets used extensively to date. It will be interesting to see whether this prediction of improved DART safety is born out as new modalities of pharmaceuticals are developed.

Having identified the HDG we then explored whether the HDG and its established MIE subset had privileged properties or locations in the HPPI as this may also aid prioritization for inclusion in a testing panel. Our community analysis showed that known MIEs are over-represented in Communities 5 and 15, and so the HDG components of these communities might be more likely to represent MIEs than those in other communities. Interestingly community 15 represents potentially new areas for research or NAM development in contrast, DARTable genes and MIEs in Communities 1 and 5 represent the signaling pathways, transcription factors described previously. Community 15, which was highly enriched for MIE nodes within the network, is overrepresented for proteins involved in substance transport and include the two main types of transporters: solute carriers (SLC), which passively transport ions across the membrane and the ATP-Binding Cassette (ABC) transporters that actively move molecules using energy from ATP (Roth et al., 2012; Walker et al., 2017). Both types of transporters are highly conserved in various species (Roth et al., 2012; Bloise et al., 2016), are potential therapeutic targets (Schumann et al., 2020) and implicated in multidrug resistance (Fletcher et al., 2016). However, they have been demonstrated to be essential for the establishment of a healthy pregnancy and are involved in transporting key substances such as steroid hormones, glucocorticoids, progesterone, fatty acids, and phospholipids across the placenta (Bloise et al., 2016; Walker, et al., 2017). ABCB1 (present in the HDG) is highly abundant in the placenta and offers fetal protection against toxicity. SLCO2A1 (which is in the HDG) is specifically involved in prostaglandin transport and is expressed in the placenta. SLCO1A2 and SLCO4A1 are not in the HDG but are in the human interactome and are involved on thyroid hormone transport (Walker et al., 2017). Whilst these transporters are in the HDG, they were annotated as either embryo lethal genes or as a developmental phenotype and not as MIEs suggesting that these proteins could be potential MIEs or could represent the link between the MIEs and the disease phenotype.

In conclusion, we have shown how bioinformatic data integration and analysis can be used to identify candidate genes that might be able to cause DART in humans: the HDG. Furthermore, our analysis has illustrated some ways in which the HDG can be a useful resource for NAM development, and so contribute to building a DART IATA. Firstly, for the prioritization and identification of MIEs within the DART AOPN, and secondly for the identification of cell lines that may be suitable screening tools to explore that AOPN within a DART IATA. And thirdly, by providing insights into relatively under-explored areas with potential for DART effects, such as the transporters proteins important for growth and development. We expect that this resource can become a common basis for further collaborative work that will enhance its utility by incorporation of other data. For example, the integration of additional cheminformatics data may allow further prioritization of MIEs. In the future a better understanding of the ligand-able proteome may be created by advances in proteome-wide structural biology and improvements in chemical docking algorithms, which would aid MIE identification. The incorporation of high content omics or imaging data can provide the experimental validation of the ability of the selected cell lines to detect the HDG MIEs. Indeed, as our original motivation was to create an optimized panel for DART screening, we will be exploring whether this panel is indeed capable of detecting the HDG MIEs. However, as there are a large number of genes (and therefore model chemicals) that need testing, we anticipate that more rapid progress would be made through a collective collaborative effort. Finally, this analysis serves as an exemplar that could be extended to detect MIE activation in other human “Toxomes” that are predictive of other toxicological endpoints such as human cancer (perhaps starting with known human cancer driver genes as seeds) and target organ toxicities. As there are many projects working on these topics across multiple industry sectors, additional collaboration to explore these topics by using as much of the accessible relevant human bioinformatic information to design future human relevant NAMs for use in IATAs. Consequently, as we continue to develop these approaches, we invite and would welcome other interested parties to contact us to further explore potential opportunities for collaboration.

## Data Availability

The original contributions presented in the study are included in the article/Supplementary Material, further inquiries can be directed to the corresponding authors.
