# Supplementary material for: Exploration of the DARTable Genome- a Resource Enabling Data-Driven NAMs for Developmental and Reproductive Toxicity Prediction
Source: Front Toxicol. 2022 Jan 19;3:806311. doi: 10.3389/ftox.2021.806311 (PMC8915813; doi:10.3389/ftox.2021.806311)
Supplement: Supplementary file 4 [file Table4.docx]

**Supplementary information 4**

Community structure definition

As indicated earlier (Barabasi, 1999), complex networks such biological systems like protein-protein interaction networks, have a scale-free distribution of nodes. In other words, in the real network we have a topology where nodes with low degree coexist with nodes with large degree. This also applies to the edge distributions in the real networks where the density of edges within particular group of nodes is higher than the average edge density in the whole network. Such group of nodes with a high density of edges within them are defined as community structures (also known as modules or clusters) (Fortunato, 2010, Fortunato and Castellano,2012), see Figure 1 reproduced from Fortunato and Castellano (2012). Each community consists of nodes that share similar properties or play a similar function in the graph. Thus, in protein-protein interaction networks proteins that are within the same community are likely to share the same specific role within the cell (Fortunato, 2010).


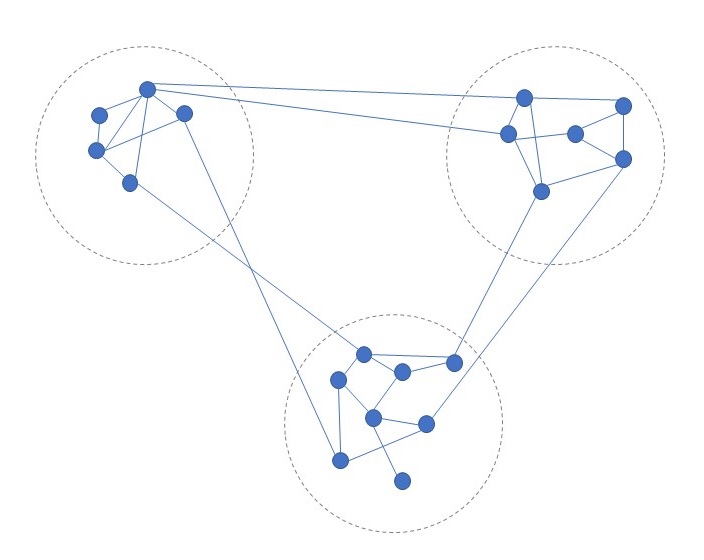


**Figure1: A simple connected component with three communities**.

Nodes and links are respectively depicted by blue circles and straight lines. Each dashed circle represents community structure (module).

Community structure detection

The community structure of the main connected component was identified by means of the greedy agglomerative algorithm knows as Louvain method (Blondel et al., 2008). The algorithm consists of two main phases: a modularity optimisation and a community aggregation. In the first phase, each node represents an individual module, Then, successive iteration takes place over all nodes to verify which vertices should be connected to increase modularity. The process is repeated until further improvement in the modularity can be obtained. In the second phase of the method, a new network is formed where the communities (modules) that have been formed in the first phase become nodes in the network and the links between those nodes are given the weight which is a sum of the weights of the links that join the two corresponding communities. Additionally, links between nodes in the same community become self-loops for this community in the new network. The step is repeated until no further gain in the modularity is achievable. As a result, it is possible to attain the best partitions of the initial network into communities.

Node role assignment – cartography analysis

A node role is characterised according to two measures adopted from the Guimera and Nunes Amaral (2005) study: within-community degree z-score and participation coefficient P. Degree z-score measures the connectivity of the node to members of the same community (module), whereas participation coefficient likewise measures its connectivity to members of other communities (modules) relative to its own module. The high value of a z-score indicates the high within-cluster node degree. The participation coefficient of a node is close to 1 if the links from a node are equally distributed among all clusters and is equal to 0 if all links of a node are within its own cluster. Based on the region in a parameter space of z-score and participation coefficient, nodes are categorised as hubs (with a higher number of links within its own community, z ≥ 2.5) and non-hubs (z < 2.5).

Non-hubs nodes are further assigned to four different roles:

R1 - ultra-peripheral node (with all links within its community (module), P ≈ 0),

R2 – peripheral node (if node has at least 60% its links within-community, 0.05 < P < 0.8)

R3 - non-hub connector node (has half of its links, or at least 2 links, whichever is larger, within community, 0.625 <P < 0.8)

R4 – non-hub kinless node (if a node has 35% of its links within-community, P > 0.8). Such nodes cannot clearly be assigned to one community. Thus, the node has links homogeneously spread among all communities.

The hub nodes, however, are divided into further three categories:

R5 – provincial hub (hub node with the great majority, at least 80%, of links within its community, P < 0.3),

R6 – connector hub (hub with many links to other clusters and at least half of its links within-community, 0.3 < P < 0.75)

R7 – global kinless hub (hub with links homogeneously spread among all clusters and fewer than half its links within-community, P > 0.75). As per R4 this identified that such nodes cannot clearly be assigned to one community.

In this study we only identified three types of non-hub nodes: R1, R2 and R3, and two types of hub nodes: R5 and R6.

Chi-square test of association

H_0_ hypothesis - there is no association between the node position in the network and its effect on node being DARTable gene.

1. Initial contingency table:

a) Observed frequencies

| **Node type** | **R1** | **R2** | **R3** | **R5** | **R6** | **Row total** |
| --- | --- | --- | --- | --- | --- | --- |
| DARTable | 436 | 2881 | 1525 | 1 | 227 | 5070 |
| NON-DARTable | 4954 | 7678 | 2841 | 11 | 239 | 15723 |
| **Colum total** | 5390 | 10559 | 4366 | 12 | 466 | 20793 |

b) Expected frequencies

| **Node type** | **R1** | **R2** | **R3** | **R5** | **R6** |
| --- | --- | --- | --- | --- | --- |
| DARTable | 1314.25 | 2574.62 | 1064.57 | 2.93 | 113.63 |
| NON-DARTable | 4075.75 | 7984.38 | 3301.43 | 9.07 | 352.37 |

Expected value is lower than 5 for R5 type node. Thus, we excluded R5 node from the test.

2. Final contingency table used for the analysis:

a) Observed frequencies

| **Node type** | **R1** | **R2** | **R3** | **R6** | **Row total** |
| --- | --- | --- | --- | --- | --- |
| DARTable | 436 | 2881 | 1525 | 227 | 5069 |
| NON-DARTable | 4954 | 7678 | 2841 | 239 | 15712 |
| **Colum total** | 5390 | 10559 | 4366 | 466 | 20781 |

b) Expected frequencies

| **Node type** | **R1** | **R2** | **R3** | **R6** |
| --- | --- | --- | --- | --- |
| DARTable | 1314.75 | 2575.60 | 1064.98 | 113.67 |
| NON-DARTable | 4075.25 | 7983.40 | 3301.02 | 352.33 |

**3. Frequency table**

| **Node type** | **DARTable node** | **O** | **E** | **O-E** | **(O-E)^2^** | **(O-E)^2^/E** |
| --- | --- | --- | --- | --- | --- | --- |
| R1 | YES | 436 | 1314.75 | -878.75 | 772209.20 | 587.34 |
| R2 | YES | 2881 | 2575.60 | 305.40 | 93268.35 | 36.21 |
| R3 | YES | 1525 | 1064.98 | 460.02 | 211622.62 | 198.71 |
| R6 | YES | 227 | 113.67 | 113.33 | 12843.93 | 112.99 |
| R1 | NO | 4954 | 4075.25 | 878.75 | 772209.20 | 189.49 |
| R2 | NO | 7678 | 7983.40 | -305.40 | 93268.35 | 11.68 |
| R3 | NO | 2841 | 3301.02 | -460.02 | 211622.62 | 64.11 |
| R6 | NO | 239 | 352.33 | -113.33 | 12843.93 | 36.45 |

O – observed frequencies, E – expected frequencies

**4. Chi-square test**

Rejected H_0_ hypothesis that there is no association between the node position in the network and its effect on node being DARTable gene.

| **ꭕ^2^** | 1236.99 |
| --- | --- |
| **α** | 0.05 |
| **df** | 3 |
| **p-value** | < 0.00001 |
| **critical value** | 7.815 |

**5. Discrepancy table**

|  | **(O-E)^2^/E** | |
| --- | --- | --- |
|  | **DARTable nodes** | **NON-DARTable nodes** |
| **R1: Ultra peripheral** | (-) 587.34 | (+) 189.49 |
| **R2: Peripheral** | (+) 36.21 | (-)11.68 |
| **R3: Non-hub connector** | (+) 198.71 | (-) 64.11 |
| **R6: Connector hub** | (+) 112.99 | (-) 36.45 |

Positive association between node type R2, R3, and R6 with DARTable nodes. Also, highly positive association between node type R1 and NON-DARTable nodes.

**References:**

Barabási, A. L., & Albert, R. (1999). Emergence of scaling in random networks. *Science*, *286*(5439), 509-512.

Fortunato S. (2010). Community detection in graphs. *Phys. Rep. Rev. Sec. Phys. Lett.* 486 75–174. 10.1016/j.physrep.2009.11.002

Fortunato S., Castellano C. (2012). Community Structure in Graphs. In: Meyers R. (eds) *Computational Complexity. Springer*, New York, NY. https://doi.org/10.1007/978-1-4614-1800-9_33
